# Sequential transcriptional changes dictate safe and effective antigen-specific immunotherapy

**DOI:** 10.1038/ncomms5741

**Published:** 2014-09-03

**Authors:** Bronwen R. Burton, Graham J. Britton, Hai Fang, Johan Verhagen, Ben Smithers, Catherine A. Sabatos-Peyton, Laura J. Carney, Julian Gough, Stephan Strobel, David C. Wraith

**Affiliations:** 1 School of Cellular and Molecular Medicine, University of Bristol, Bristol BS8 1TD, UK; 2 Computational Genomics Group, Department of Computer Science, University of Bristol, Bristol BS8 1UB, UK; 3 Division of Biomedical Sciences, Institute of Child Health, University College London, London WC1N 1EH, UK

## Abstract

Antigen-specific immunotherapy combats autoimmunity or allergy by reinstating immunological tolerance to target antigens without compromising immune function. Optimization of dosing strategy is critical for effective modulation of pathogenic CD4^+^ T-cell activity. Here we report that dose escalation is imperative for safe, subcutaneous delivery of the high self-antigen doses required for effective tolerance induction and elicits anergic, interleukin (IL)-10-secreting regulatory CD4^+^ T cells. Analysis of the CD4^+^ T-cell transcriptome, at consecutive stages of escalating dose immunotherapy, reveals progressive suppression of transcripts positively regulating inflammatory effector function and repression of cell cycle pathways. We identify transcription factors, c-Maf and NFIL3, and negative co-stimulatory molecules, LAG-3, TIGIT, PD-1 and TIM-3, which characterize this regulatory CD4^+^ T-cell population and whose expression correlates with the immunoregulatory cytokine IL-10. These results provide a rationale for dose escalation in T-cell-directed immunotherapy and reveal novel immunological and transcriptional signatures as surrogate markers of successful immunotherapy.

## Abstract (web summary)

Dose escalation in antigen-specific therapies is recognized as safe and effective, but the underlying effects of dosing variables on the immune system are not understood. Here, the authors demonstrate that dose escalation causes sequential modulation of gene expression among antigen-specific lymphocytes.

## Graphical abstract

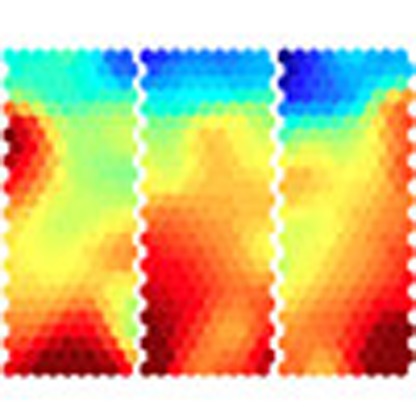

Autoimmune diseases are a diverse group of chronic inflammatory conditions affecting millions of individuals worldwide and are caused by an inappropriate immune response mounted against the body’s own tissues. While progress has been made in developing disease-modifying therapies for the treatment of autoimmunity, it is increasingly clear that successful therapy will need to reinstate long-lasting immunological tolerance to the targeted self-antigens^1^,^2^, thereby preventing pathogenic CD4^+^ T-cell responses. This must be achieved without perturbation of normal immune function, leaving anti-microbial and tumour immunosurveillance responses intact. Antigen-specific immunotherapy aims to fulfil these requirements: administration of disease-associated CD4^+^ T-cell epitopes in a tolerogenic form has been shown to restore immune homeostasis and prevent immunopathology in experimental models^3^,^4^,^5^, as well as in clinical trials of both autoimmune diseases^6^,^7^,^8^ and allergies^9^,^10^,^11^.

It is clear that regulatory CD4^+^ T cells play an integral role in the success of this approach^1^; yet, we still lack a comprehensive understanding of the mechanisms underlying therapeutic development of antigen-specific tolerance. Induction of the pleiotropic, immunomodulatory cytokine interleukin (IL)-10 is frequently associated with efficacious peptide immunotherapy in both mouse and man^8^,^12^,^13^,^14^. In the experimental autoimmune encephalomyelitis model (EAE) of multiple sclerosis, intranasal (i.n.) administration of a soluble myelin basic protein (MBP) peptide induces tolerance^15^,^16^,^17^ through the induction of IL-10-secreting CD4^+^ FoxP3^-^ T cells^16^,^17^,^18^,^19^. During the course of immunotherapy, chronic stimulation of CD4^+^ T cells by repetitive i.n. peptide administration culminates in an altered transcriptional programme^20^, with pathogenic Th1 cells driven to an anergic, IL-10-secreting, regulatory phenotype^21^ capable of preventing autoimmunity. Induction of IL-10 expression by self-reactive CD4^+^ T cells is consequently a highly desirable therapeutic goal.

In the clinic, allergen-specific immunotherapy typically involves administration of escalating doses of antigen in the early phase of treatment, before a high maintenance dose is reached, resulting in allergic desensitization^1^,^22^. It is widely accepted that use of dose escalation strategies minimizes the risk of immunotherapy-associated adverse effects, which may range from mild symptoms to anaphylaxis. Dose escalation permits administration of larger antigen doses and, when successful, the reinstatement of immunological tolerance towards the administered antigen. Despite this long-held consensus, the molecular and immunological changes that occur during the escalation phase of treatment to modulate the immune response are poorly understood. Many factors influence the outcome of antigen-specific immunotherapy using either self- or non-self-antigens. These include the form of the chosen antigen (protein versus peptide), antigen dose and frequency of administration^23^. The challenge of developing this targeted approach for the treatment of autoimmune disease lies not only in appreciating the effect that these dosing variables have on the clinical outcome of immunotherapy, but also in gaining a deeper understanding of the processes underlying effective antigen-specific immunotherapy. By better understanding these processes, we will be able to refine and enhance therapeutic tolerance induction, minimizing treatment-associated risks, and achieving sustained modulation of pathogenic antigen-specific CD4^+^ T-cell activity.

In light of these considerations, we have developed a dose escalation strategy for efficient self-antigen-specific tolerance induction via a non-mucosal route, and characterized sequential modulation of CD4^+^ T-cell phenotype at each consecutive stage of escalating dose immunotherapy (EDI). Here, using the Tg4 T-cell receptor (TCR) transgenic model of EAE to study antigen-specific CD4^+^ T-cell responses^15^, we show that self-antigen-specific tolerance can be effectively induced via the subcutaneous (s.c.) route, eliciting IL-10-secreting CD4^+^ T cells with an anergic, regulatory phenotype. We demonstrate that antigen dose plays a critical role in determining the efficacy of immunotherapy, and that a dose escalation protocol is imperative to allow safe s.c. administration of the high antigenic doses required for efficient tolerance induction. We reveal that EDI minimizes CD4^+^ T-cell activation and proliferation during the early stages of immunotherapy, preventing excessive systemic cytokine release. Notably, tolerance induced by EDI is effective in EAE models whether administered prophylactically or therapeutically. We demonstrate the CD4^+^ T-cell transcriptome-wide changes at sequential stages of EDI, applying an unbiased bioinformatics approach to identify groups of transcripts with co-ordinated expression, and infer the biological significance of these co-ordinated expression patterns. We identify the sequential induction of signature transcription factors and negative co-stimulatory molecules, which characterize the CD4^+^ T-cell population induced during therapeutic tolerance induction. These findings reveal the critical importance of dose escalation in the context of antigen-specific immunotherapy, as well as the immunological and transcriptional signatures associated with successful self-antigen EDI.

## Results

### Tolerance induction is dose dependent

**Figure 1:**
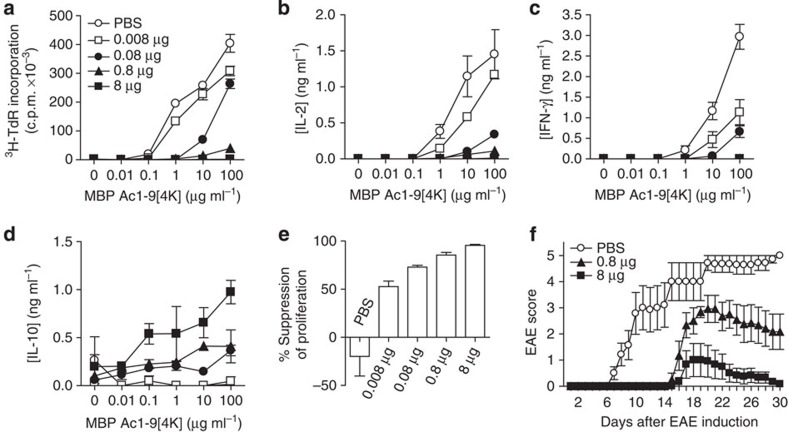
Peptide dose dictates regulatory CD4^+^ T-cell phenotype and protection from EAE. CD4^+^ T cells from Tg4 mice treated 10 times s.c. with different doses of MBP Ac1-9[4Y] were re-stimulated in the presence of irradiated antigen-presenting cells (APCs) and a titration of MBP Ac1-9[4K]. (**a**) After 3 days, proliferative responses were measured by ^3^[H] thymidine incorporation. (**b**–**d**) Cytokines detected in cultures described in **a**, measured by an enzyme-linked immunosorbent assay (ELISA). (**e**) *In vitro*-expanded CD4^+^ T cells from peptide-treated Tg4 mice were cultured at a ratio of 1:1 with responder CD4^+^ T cells from untreated Tg4 mice, irradiated APCs and 0.1 μg ml^−1^ MBP Ac1-9[4K]. After 3 days, proliferative responses were measured by ^3^[H] thymidine incorporation. Graph shows percentage suppression of responder CD4^+^ T-cell proliferation, relative to proliferation of responder cells cultured alone. Data (**a**–**e**) are representative of three similar experiments, error bars show s.e.m. of two independent biological replicates, each assayed in triplicate. (**f**) Onset and severity of EAE in Tg4 mice pre-treated with 10 doses of MBP Ac1-9[4Y] before immunization with spinal cord homogenate/Complete Freund's Adjuvant and Pertussis toxin. Results of two independent experiments are pooled, showing mean disease score±s.e.m. (*n*=7 PBS group, *n*=6 for each peptide-treated group).

We have investigated the s.c. route for self-peptide immunotherapy in the Tg4 TCR transgenic model of EAE^15^, where >90% of CD4^+^ T cells recognize the nine-residue N-terminal peptide of MBP (MBP Ac1-9). The dose of a high-affinity major histocompatibility complex binding MBP peptide (MBP Ac1-9[4Y])^24^ required for effective tolerance induction by this route was determined. The proliferative capacity of CD4^+^ T cells was reduced in direct correlation to the MBP Ac1-9[4Y] dose administered *in vivo* (Fig. 1a). While proliferation of CD4^+^ T cells from animals treated with the lowest peptide dose (0.008 μg) remained unaltered, CD4^+^ T cells from mice treated at a higher (8 μg) dose displayed proliferative anergy. Abrogation of the secretion of Th1-associated cytokines, IL-2 (Fig. 1b) and interferon (IFN)-γ (Fig. 1c), was also observed among CD4^+^ T cells from mice treated with 8 μg MBP Ac1-9[4Y]. A direct correlation was noted between IL-10 secretion and peptide dose (Fig. 1d); IL-10 levels were negligible from control or 0.008 μg treatment groups, while CD4^+^ T cells from mice treated with 8 μg secreted the highest level. A dose-dependent effect on the development of a suppressive phenotype was observed *in vitro* (Fig. 1e). CD4^+^ T cells from animals treated with 0.8 and 8 μg doses suppressed responder cell proliferation by 85% and 95%, respectively. *In vivo*, treatment of Tg4 mice with repetitive 0.8 or 8 μg doses of MBP Ac1-9[4Y] delayed the onset and reduced the severity of myelin-induced EAE in a dose-dependent manner (Fig. 1f).

### Inflammatory cytokines are induced at high antigen doses

**Figure 2:**
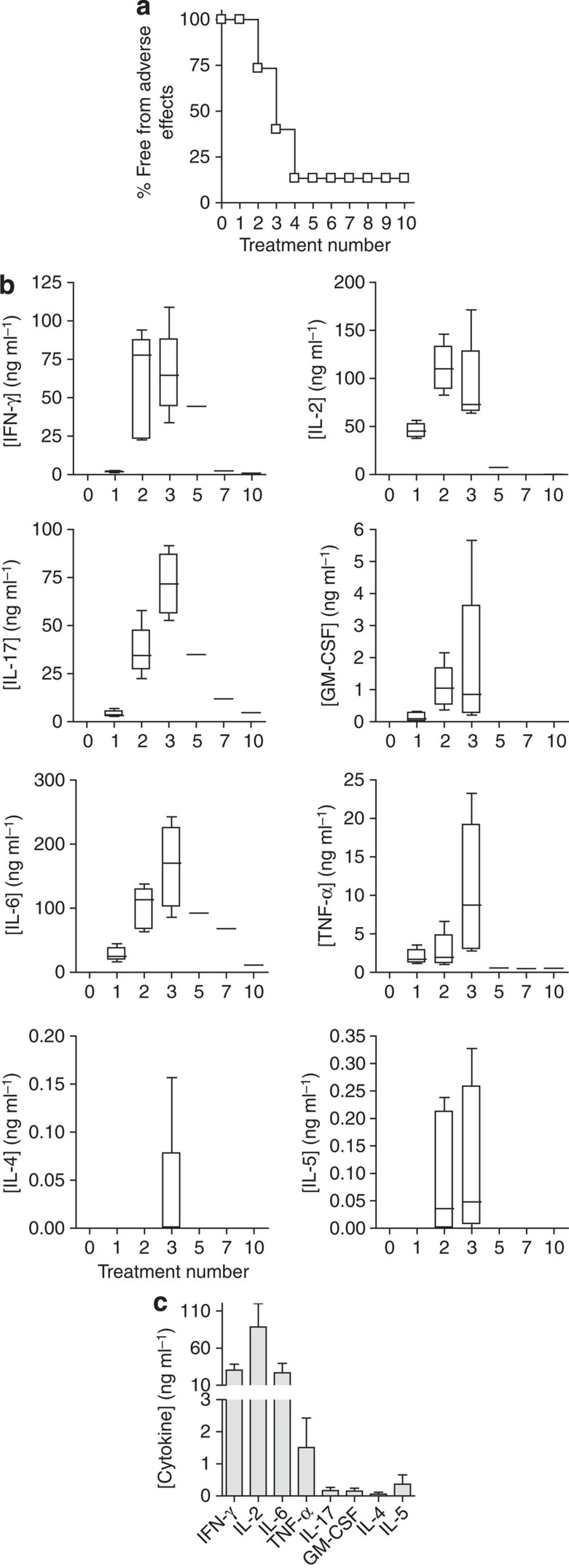
High peptide doses administered subcutaneously induce inflammatory cytokines in TCR transgenic mice. Tg4 mice were treated 10 times s.c. with 80 μg MBP Ac1-9[4Y]. (**a**) Shows the percentage of mice (*n*=15) free from adverse effects during the course of treatment. (**b**) Box-and-whisker plots show cytokine concentrations in serum (*n*=6), collected 2 h after successive 80 μg MBP Ac1-9[4Y] treatments as detected by a multiplex fluorescent bead immunoassay (MFBI). Box extends from 25th to 75th percentiles, horizontal line in boxes represents median value, error bars show minimum and maximum values. Data are representative of three independent experiments. (**c**) MFBI-detected serum cytokines from Tg4 Rag-1^−/−^ mice 2 h after a second s.c. injection with 80 μg MBP Ac1-9[4Y]. Mean cytokine concentration+s.e.m. is shown for four individual animals.

A higher dose of MBP Ac1-9[4Y] (80 μg) has been used to treat Tg4 mice by the i.n. route^21^. However, this dose was not tolerated when administered s.c. (Fig. 2a). Adverse effects (hunched posture, reduced mobility and responsiveness, piloerection and dyspnoea) developed between 2 and 24 h after the second and third injections. High concentrations of inflammatory cytokines were detected in the serum of treated mice, whereas Th2 cytokine concentrations remained low (Fig. 2b). Immunoglobulin E-mediated anaphylaxis has been reported to follow the administration of soluble myelin peptides after induction of EAE^25^,^26^. We noted, however, that adverse effects concomitant with high inflammatory cytokine levels developed similarly in the B-cell-deficient Tg4 Rag-1^−/−^ mouse (Fig. 2c), suggesting that high-dose complications observed in the TCR transgenic Tg4 model were not antibody mediated, but a consequence of an excessive CD4^+^ T-cell response.

### Dose escalation is critical for effective immunotherapy

**Figure 3:**
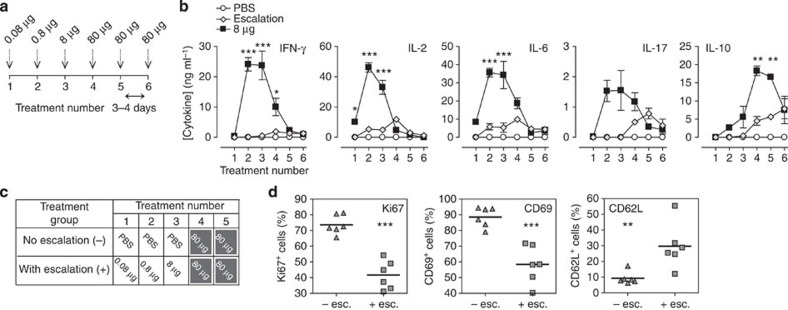
Peptide dose escalation desensitizes antigen-specific CD4^+^ T cells, allowing safe administration of high peptide doses subcutaneously. (**a**) MBP Ac1-9[4Y] dose escalation strategy. (**b**) Mean serum cytokine concentrations, detected by MFBI, 2 h after treatment of Tg4 mice with either six 8-μg MBP Ac1-9[4Y] doses or EDI (illustrated in **a**). Representative of three experiments, error bars show±s.e.m. of three biological replicates. *P≤0.05, **P≤0.01, ***P≤0.001, two-way analysis of variance with Bonferroni post-test, comparing 8-μg MBP Ac1-9[4Y]- and EDI-treated mice. Tg4 mice were treated s.c. with high antigen doses, with (+) or without (−) prior dose escalation (dosing strategy illustrated in **c**). CD4^+^ T-cell expression of Ki67, CD69 and CD62L 2 h after peptide challenge *in vivo* was determined by flow cytometric analysis (**d**). Scatter plots show the percentage of CD4^+^ cells from individual mice, which are Ki67^+^, CD69^+^ or CD62L^+^, horizontal lines show means for each column. Results of two independent experiments are pooled (*n*=6). **P≤0.01, ***P≤0.001, unpaired *t*-test.

We determined whether escalating doses (0.08 μg→0.8 μg→8 μg→3 × 80 μg) would prevent the adverse effects associated with a high-dose regimen (Fig. 3a). No adverse effects were seen in EDI-treated animals despite the high dose of peptide given. Serum cytokine concentrations were measured at each stage of EDI (Fig. 3b) and were compared with mice treated with a constant 8 μg Ac1-9[4Y], the highest safe s.c. dose investigated. While an early peak in inflammatory cytokine levels was detected in mice treated with a constant 8 μg dose, the response was significantly lower (IFN-γ, IL-2 and IL-6) or much delayed (IL-17) in the EDI group. Levels of IL-10 were found to be similar at the end of both treatment courses, indicating that subversion of the initial inflammatory response did not compromise induction of the anti-inflammatory cytokine.

Next we investigated the mechanism by which EDI permits s.c. administration of high, 80 μg, MBP Ac1-9[4Y] doses without adverse effects. Tg4 mice were treated with two high, 80 μg, MBP Ac1-9[4Y] doses, with or without prior dose escalation (Fig. 3c). The proliferative and activation status of antigen-specific CD4^+^ T cells was determined after the second 80-μg dose (Fig. 3d). CD4^+^ T cells from EDI-treated mice showed significantly lower expression of Ki67, a protein associated with cell division, than animals treated without dose escalation. Expression of the activation marker CD69 was also significantly suppressed in EDI-treated mice, while CD62L expression was significantly higher. These results demonstrate that dose escalation desensitises self-antigen-specific CD4^+^ T cells, suppressing activation and proliferation in response to cognate antigen, even at high doses.

**Figure 4:**
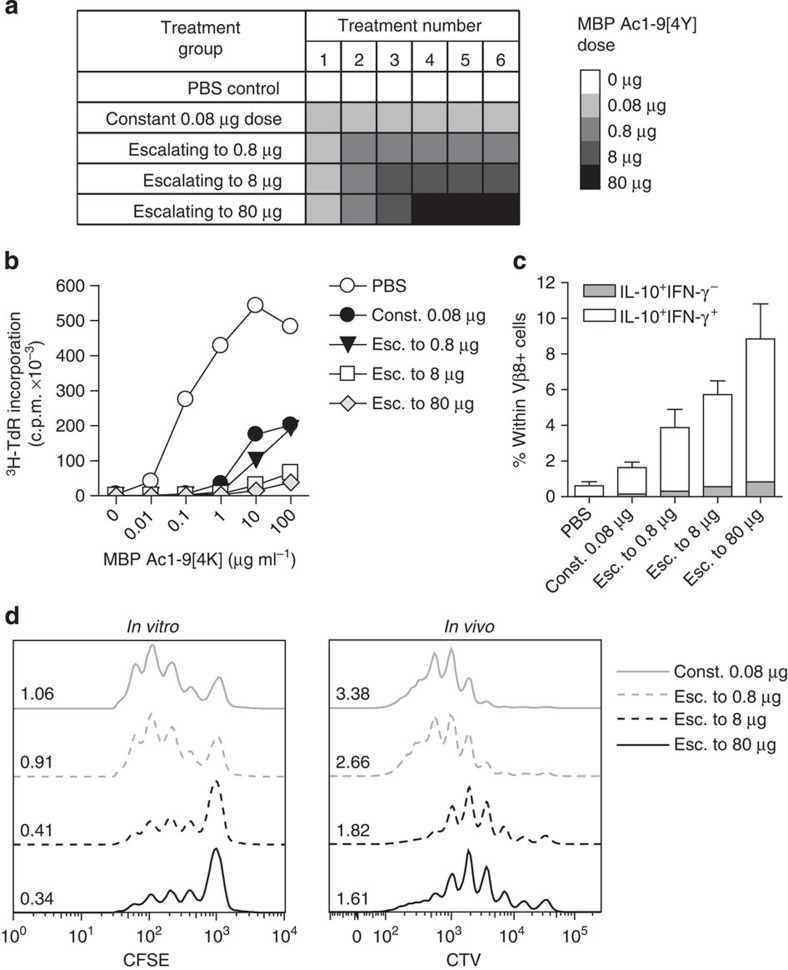
Dose escalation to higher peptide doses improves induction of a regulatory CD4^+^ T-cell phenotype. (**a**) Treatment groups for EDI with increasingly higher final doses. (**b**) Proliferative responses of CD4^+^ T cells from EDI-treated Tg4 mice cultured with irradiated antigen-presenting cells (APCs) and MBP Ac1-9[4K] for 3 days, measured by ^3^[H] thymidine incorporation. Representative of three independent experiments each with two biological replicates assayed in triplicate. (**c**) Percentages of Vβ8^+^ T cells expressing IL-10 and IFN-γ after 6 days culture with 10 μg ml^−1^ MBP Ac1-9[4K], detected by flow cytometric analysis. Representative of two similar experiments, error bars show+s.e.m. of two biological replicates. (**d**) For *in vitro* suppression assay, *in vitro*-expanded CD4^+^ T cells from peptide-treated Tg4 mice cultured 1:1 with carboxyfluorescein succinimidyl ester (CFSE)-labelled responder CD4^+^ T cells, APCs and 10 μg ml^−1^ MBP Ac1-9[4K]. After 3 days, the proliferative response of CD4^+^CFSE^+^ responder cells was measured by flow cytometry. For *in vivo* suppression assay, 5 × 10^6^ Cell Trace Violet (CTV)-labelled CD45.1^+^ Tg4 CD4^+^ T cells were transferred i.v. into EDI-treated Tg4 CD45.2^+^ mice. After 24 h, mice were injected s.c. with 80 μg of MBP Ac1-9[4Y]. Three days after peptide challenge, Cell Trace Violet-labelled CD45.1^+^ CD4^+^ cells were recovered from spleens for flow cytometric analysis. Data in each plot are representative of two biological replicates, offset histograms show proliferation dye dilution and division indexes.

**Figure 5:**
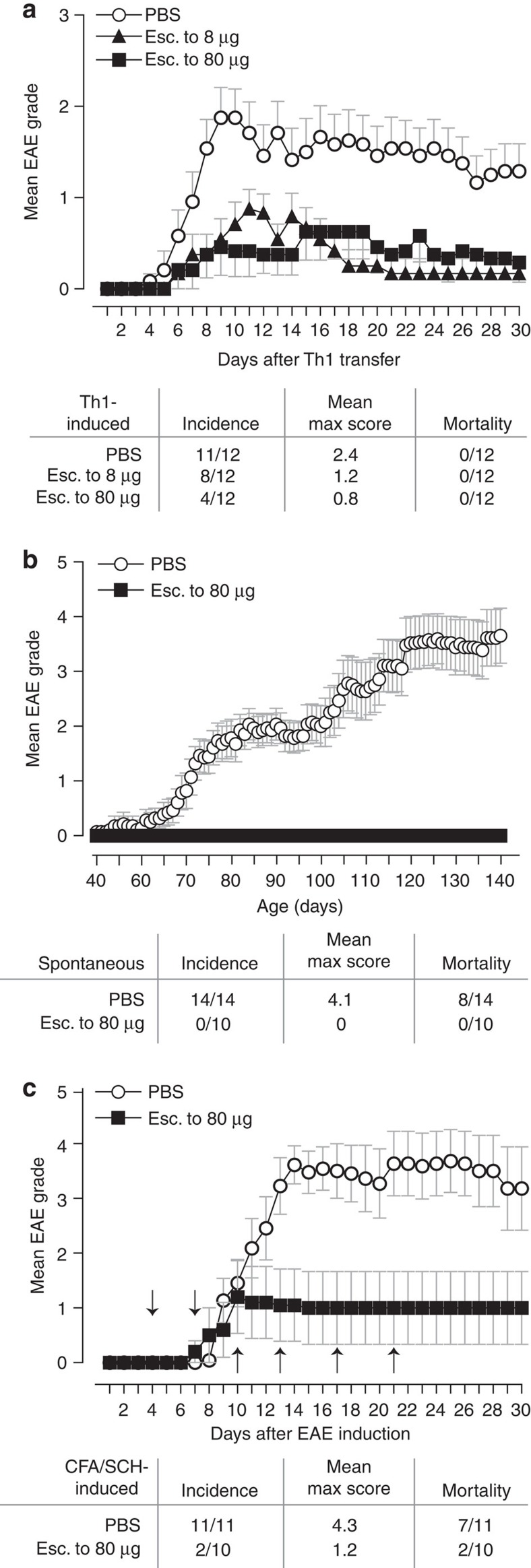
Escalating dose immunotherapy reduces EAE susceptibility. (**a**) Tg4 mice were pre-treated s.c. with an escalating course of MBP Ac1-9[4Y], either 0.08 μg→0.8 μg→4 × 8 μg (esc. to 8 μg) or 0.08 μg→0.8 μg→8 μg→3 × 80 μg (esc. to 80 μg). *In vitro*-differentiated MBP Ac1-9-specific Th1 cells (10^7^) were transferred i.v. to treated animals. (**b**) Six-week-old Tg4 Rag-1^−/−^ mice, susceptible to spontaneous development of EAE, were EDI-treated (esc. to 80 μg, as above). (**c**) EAE was induced in Tg4 mice by s.c. injection of Complete Freund's Adjuvant (CFA)/spinal cord homogenate (SCH) with Pertussis toxin given on day 0 and day 2. Animals were then EDI-treated (arrows indicate day of treatment, dosing as above escalating to 80 μg). Animals were monitored daily for onset of EAE symptoms. Graphs show mean EAE score and s.e.m., summary panel shows incidence, mean maximum score and mortality for each experiment, *n* as shown in **a**–**c**.

A strong TCR signal is required for the development of IL-10-secreting CD4^+^ T cells^17^,^27^,^28^,^29^,^30^. The outcome of altering the end-point dose in EDI was investigated, using EDI with increasingly higher final doses (Fig. 4a). CD4^+^ T cells from all peptide-treated groups displayed a dose-dependent reduction in proliferative capacity (Fig. 4b), concomitant with an increase in the proportion of IL-10^+^IFN-γ^+^ T cells (Fig. 4c). Furthermore, the suppressive capacity of CD4^+^ T cells induced by EDI, either *in vitro* or *in vivo*, correlated with the magnitude of the final dose administered (Fig. 4d). These results reveal the direct correlation between peptide dose and the induction of a regulatory phenotype in auto-reactive T cells. Experiments were undertaken to determine whether the escalation phase of EDI itself enhances the induction of tolerance by EDI, in addition to reducing the risk of adverse effects in response to treatment. This was done by comparing the effects of repetitive 8-μg doses of MBP Ac1-9[4Y], with or without prior dose escalation (Supplementary Fig. 1a). The percentage of IL-10^+^ CD4^+^ T cells was higher in animals treated with prior dose escalation (Supplementary Fig. 1b); however, resistance to EAE was comparable in both of these treatment groups (Supplementary Fig. 1c). The robustness of tolerance induced by EDI was investigated *in vivo* using three disease models, testing both the prophylactic and therapeutic efficacy of EDI. Tg4 recipients pre-treated with EDI demonstrated reduced disease incidence following adoptive transfer of *in vitro*-differentiated MBP Ac1-9-specific Th1 cells (Fig. 5a). Tg4 Rag-1^−/−^ mice lack FoxP3^+^ nTreg cells and develop EAE spontaneously^19^. EDI treatment of healthy 6-week-old Tg4 Rag-1^−/−^ mice provided complete protection from spontaneous EAE, up to and beyond 20 weeks of age (Fig. 5b). Therapeutic treatment of Tg4 mice after EAE induction with Complete Freund's Adjuvant (CFA)/spinal cord homogenate and Pertussis toxin reduced disease incidence and mortality in EDI-treated mice, indicating that EDI is effective in both naive and primed settings (Fig. 5c).

### Progressive CD4^+^ T-cell transcriptome changes during EDI

**Figure 6:**
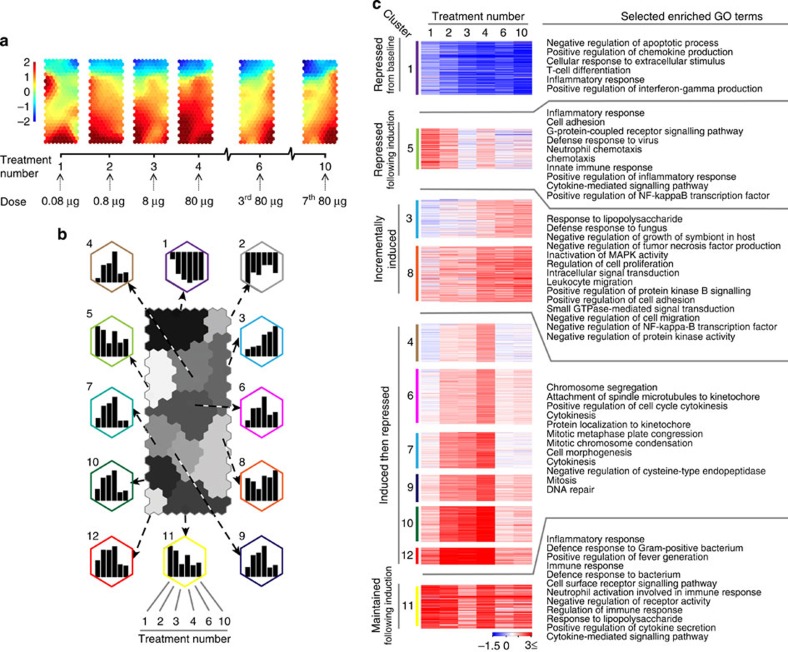
Transcriptome analysis of CD4^+^ T cells at consecutive stages of escalating dose immunotherapy. Tg4 Rag-1^−/−^mice were EDI-treated s.c. with MBP Ac1-9[4Y]. CD4^+^ T-cell transcriptome analysis was undertaken at the indicated treatment stages (*n*=3 per time point, RNA pooled). (**a**) CPP-SOM of 1,893 regulated transcripts across six stages of EDI, illustrating treatment stage-specific transcriptional changes, relative to PBS-treated controls. Hexagons represent groups of co-clustered transcripts; colour changes show modulation of transcript expression (upregulation in red, downregulation in blue and moderate regulation in yellow and green). (**b**) Twelve gene clusters (colour-coded, labelled 1–12) obtained by two-phase SOM clustering. Bar graphs show the expression pattern of the seed unit used to derive each gene cluster. (**c**) Heatmaps showing expression patterns of clustered genes, and GO terms associated with each. Gene clusters are organized according to five dominant patterns; genes that are repressed from baseline, repressed following initial induction, incrementally induced, induced and then repressed and finally, induced and then maintained throughout the course of treatment. All GO terms are associated with at least three transcripts within a cluster, with a false-discovery rate (FDR) of <0.05.

Whole-genome expression arrays were used to generate transcriptome data of CD4^+^ T cells at successive stages of EDI. On the basis of the expression matrix of 1,893 transcripts regulated across six stages of EDI (Supplementary Table 1), we applied a self-organizing map (SOM)-based method for gene clustering and visualization^31^. Treatment stage-specific transcriptome changes during EDI are illustrated using component plane presentations (CPPs)^32^ (Fig. 6a). Comparison of presentations reveals that, while dynamic changes occur during the initial stages of treatment, the transcriptional profile induced by extended high doses (between treatments 6 and 10) remains remarkably stable. A second phase of unbiased analysis was undertaken, yielding 12 clusters on a SOM-CPP grid along with the representative expression pattern of each (Fig. 6b; Supplementary Table 1). To elucidate the functional relevance of transcripts within these gene clusters (excluding cluster 2 with only nine transcripts), we performed enrichment analysis to identify gene ontology-biological process (GO-BP)^33^ terms that were significantly associated with these expression patterns (Fig. 6c; Supplementary Table 2). Genes that were repressed, either from baseline (cluster 1) or following induction (cluster 5), were enriched with GO-BP terms associated with the induction of an inflammatory response. Genes relating to regulatory immune processes were incrementally induced during EDI (patterns in clusters 3 and 8), including negative regulation of MAPK, NFκB and protein kinase signalling pathways. The genes of cluster 11, associated with the inflammatory response and cytokine production, were upregulated upon the first exposure to self-antigen and broadly maintained throughout the course of immunotherapy. Many genes (those in clusters 4, 6, 7, 9, 10 and 12) were upregulated during the escalation phase of treatment, then strongly repressed following extended high doses. Terms enriched within this pattern were strongly associated with cell cycle-related processes, such as mitosis, cytokinesis and microtubule regulation. Additional enrichments using Mouse Genome Informatics-Mammalian Phenotype^34^, Reactome^35^ and Disease Ontology (DO)^36^ terms (Supplementary Fig. 2; Supplementary Table 2) supported that clusters of genes sharing this expression pattern were related to cell cycle processes and diseases of abnormal cell division. Interrogation of the DO database also revealed significant association of terms describing inflammatory autoimmune diseases within cluster 5, where gene expression is repressed following initial exposure to the antigen.

**Figure 7:**
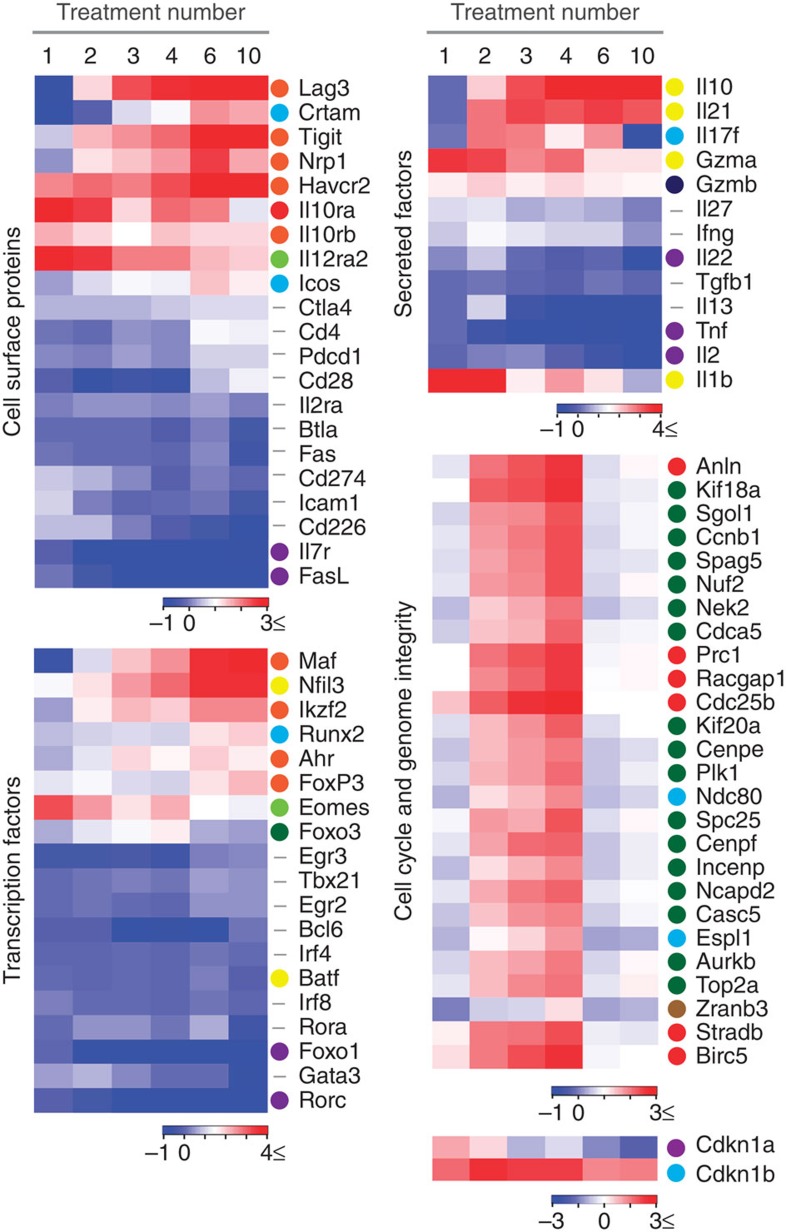
Modulated expression of select genes associated with regulatory T-cell phenotype during escalating dose immunotherapy. Tg4 Rag-1^−/−^ mice were EDI-treated s.c. with MBP Ac1-9[4Y]. CD4^+^ T-cell transcriptome analysis was undertaken at the indicated treatment stages (*n*=3 per time point, RNA pooled) and transcripts were grouped by two-phase SOM clustering (see Fig. 6). Heatmaps are used to illustrate the fold changes in mRNA expression of individual genes during EDI. The cluster from which individual genes are derived (illustrated in Fig. 6b) is indicated by a colour identifier. Genes that were not included in clustering analysis (did not demonstrate a two-fold or greater change at ≥;4 treatment points, compared with the PBS-treated control) are indicated by a hyphen.

On the basis of the expression patterns and their functional relevance revealed above, we selected genes characterizing the altered CD4^+^ T-cell state during the course of EDI (Fig. 7). Expression of negative co-stimulatory molecules including *Lag3*, *Tigit* and *Havcr2* (TIM-3), were highly upregulated during treatments 1–6 of EDI and were maintained with extended treatment. Conversely, expression of *Pdcd1* (PD-1) and *Ctla4* remained largely unchanged during treatment, while expression of *Btla* and *Cd274* (PD-L1) decreased with extended treatment. *Icos*, reported to promote IL-10 expression in CD4^+^ T cells^37^,^38^, was upregulated, while expression of the positive co-stimulatory molecule *Cd226* decreased with treatment. As anticipated, expression of *Il10* rose incrementally during EDI, a pattern shared with *Il21*, a cytokine previously shown to act as an autocrine growth factor of IL-10-secreting T cells^38^. The transcription factors c-Maf and NFIL3 (also known as E4BP4) are both implicated in regulation of IL-10 production by CD4^+^ T cells^38^,^39^; *Maf* and *Nfil3* transcript expression was highly upregulated during EDI. Expression of *Ahr*, a transcription factor that interacts with c-Maf for transactivation of the IL-10 and IL-21 promoters^40^, followed this trend at a lower magnitude. Transcripts pertaining to cell cycle and genome integrity were induced during the escalation phase of treatment and then repressed with extended high doses. These included regulators of mitotic spindle dynamics and G2/M-phase transition, as well as spindle checkpoint components. Aurora kinase B (*Aurkb*), survivin (*Birc5*) and *Incenp*, previously associated with cell cycle progression in T cells^41^, were all repressed following extended high-dose treatment. In contrast to published studies^42^, both of the anergy-associated cyclin-dependant kinase inhibitors, p21^Cip1^ (*Cdkn1a*) and p27^Kip1^ (*Cdkn1b*), were repressed during the course of EDI. Together, these results highlight that each escalating dose of peptide treatment modifies the CD4^+^ T-cell transcriptome in a co-ordinated manner, resulting in a distinct EDI-induced phenotype characterized by the expression of specific transcription factors, negative co-stimulatory molecules and cytokines.

### Incremental induction of a regulatory CD4^+^ T-cell phenotype

**Figure 8:**
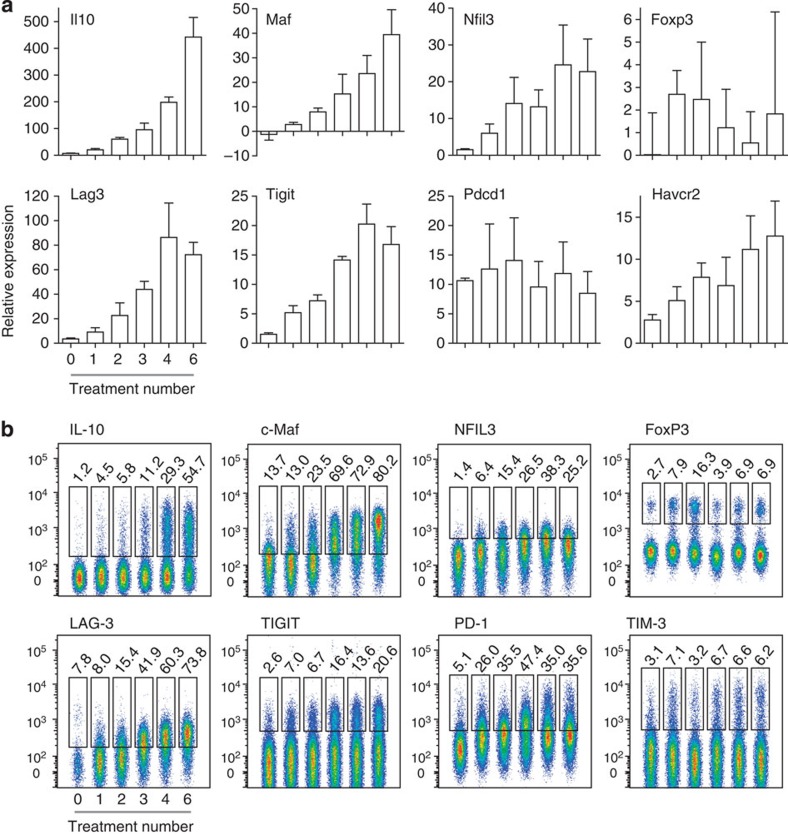
CD4^+^ T-cell signature induced by dose escalation immunotherapy. Tg4 mice were treated s.c. with an escalating dose of MBP Ac1-9[4Y] (0.08 μg→0.8 μg→8 μg→3 × 80 μg). (**a**) Real-time PCR analysis of mRNA of select genes expressed by CD4^+^ T cells, 2 h after peptide challenge *in vivo*, at the indicated stages of treatment. Graphs show mean expression values+s.e.m. for three replicate experiments, pooled (*n*=3 total). (**b**) Flow cytometric staining of IL-10, c-Maf, NFIL3, FoxP3, LAG-3, TIGIT, PD-1 and TIM-3 by CD4^+^ T cells at the indicated stages of treatment. Cells were harvested 2 h after peptide challenge *in vivo*. Data are representative of two to three independent experiments.

**Figure 9:**
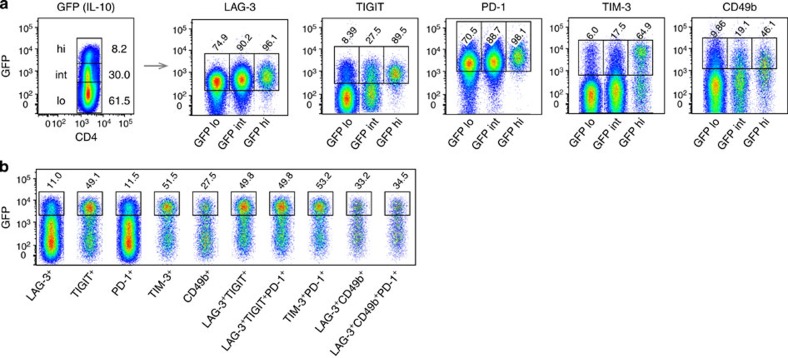
Correlation between IL-10 and expression of surface molecules associated with a regulatory T-cell phenotype. Tg4^GFP/Il10^ mice were treated with an escalating dose of MBP Ac1-9[4Y] (0.08 μg→0.8 μg→8 μg→3 × 80 μg), inducing GFP(IL-10) low (lo), intermediate (int) or high (hi) expression by CD4^+^ T cells. (**a**) Percentages of GFP(IL-10) lo, int and hi cells expressing LAG-3, TIGIT, PD-1,TIM-3 and CD49b at the cell surface. (**b**) Expression of GFP(IL-10) by CD4^+^ T cells expressing the indicated cell surface markers. Data are representative of nine mice, over three independent experiments.

Expression of selected genes characterizing the CD4^+^ T-cell transcriptional state induced by EDI was confirmed by real-time PCR (Fig. 8a). This supports the incremental induction of transcripts for transcription factors and negative co-stimulatory molecules commonly associated with T-cell tolerance, as well as IL-10. CD4^+^ T-cell expression of these markers at the protein level was confirmed (Fig. 8b; Supplementary Fig. 3). Similar patterns of protein expression were observed in resting (Supplementary Fig. 4) and recently activated cells (Fig. 8b), although expression was higher in activated T cells. Indeed, a gradual increase in NFIL3 expression was detected only in activated cells. The percentage of CD4^+^ T cells expressing IL-10, c-Maf or LAG-3 expanded sequentially during EDI, culminating in at least 50% of activated cells expressing these markers. A rising percentage of TIGIT^+^ cells also accumulated during EDI (20% of activated CD4^+^ T cells). The proportion of cells expressing TIM-3 remained relatively stable throughout EDI, while the percentage of PD-1^+^ cells increased upon initial CD4^+^ T-cell activation and further increased during the later stages of EDI. An early peak in the percentage of FoxP3^+^ cells was detected, following low-dose treatment, but the percentage of FoxP3^+^ cells did not correlate with effective tolerance induction. Although expression of *Il21* messenger RNA (mRNA) increased sequentially throughout the course of EDI, no change in CD4^+^ T-cell expression of IL-21 at the protein level was detected (Supplementary Fig. 5). We generated Tg4^Il10/GFP^ reporter mice by crossing Tg4 mice with B6.129S6-Il10^tm1Flv^/J mice, originally generated and characterized by Kamanaka *et al*.^43^ Tg4^Il10/GFP^ reporter mice were then used to determine the correlation between negative co-stimulatory molecule expression and IL-10. CD4^+^ T cells from treated mice were divided on the basis of green fluorescent protein (GFP) (IL-10) expression (Fig. 9a). Levels of LAG-3, TIGIT, PD-1 and TIM-3 correlated positively with IL-10 in CD4^+^ T cells, as did expression of CD49b, a marker recently used in combination with LAG-3 for the detection of IL-10-secreting Tr1 cells^44^. However, GFP^−^(IL-10^−^) cells were also detected among populations demonstrating high-level expression of these markers (Fig. 9b).

## Discussion

The aim of this study was to define an optimal strategy for safe and effective antigen-specific immunotherapy. Variables for this approach include the nature of the antigen, dose and route of administration. As reviewed elsewhere^23^,^45^, peptide epitopes targeting CD4^+^ T cells have distinct advantages over intact antigens, and yet the mechanism by which peptide therapy prevents and treats ongoing autoimmune and allergic diseases is poorly defined. Mucosal routes of administration have proven safe and effective in animal models of allergy and autoimmunity, but have not translated well to the clinic. Here we demonstrate that the s.c. route of administration is more effective than the i.n. route, with a 1,000-fold lower dose of antigen being effective for anergy induction when compared with previous studies^17^,^18^. As noted^17^, the efficacy of tolerance induction and disease prevention depends on signal strength. In this study, all aspects of inflammatory T-cell function, including proliferation, inflammatory cytokine secretion and encephalitogenicity were suppressed, whereas the ability of cells to secrete IL-10 and suppress EAE increased in a dose-dependent manner. IL-10 clearly serves as a promising mediator of effective antigen-specific immunotherapy^1^,^12^.

The dose of peptide previously shown to be safe and effective for i.n. tolerance (80 μg MBP Ac1-9[4Y]) led to high levels of inflammatory cytokines when administered s.c. in Tg4 mice, presumably because of the high frequency of antigen-specific T cells. The high cytokine release elicited by administration of repeated high-dose antigen in this TCR transgenic model is reminiscent of previous attempts to modulate the immune response with antibodies against TCR or CD28. Anti-CD3 treatment has been associated with toxic shock^46^, while anti-CD28 caused elevated cytokine release^47^. We reasoned that excessive CD4^+^ T-cell activation would be avoided by initiating peptide treatment at a lower dose, since even a 0.08-μg dose was able to induce anergy when administered by the s.c. route. By using the EDI protocol, it was possible to reach the high doses of antigen required for IL-10 induction without the risk of elevated inflammatory cytokines. The phenotype of CD4^+^ T cells induced by EDI was comparable to that of cells induced by repetitive administration of a constant 8-μg dose of MBP Ac1-9[4Y]. In both cases, cells were anergic, suppressive and expressed IL-10. Experiments comparing IL-10 expression by CD4^+^ T cells from mice treated with three 8-μg doses of peptide with or without prior dose escalation showed higher IL-10 in animals treated with prior dose escalation. This suggests that the escalation phase itself enhances tolerance induction, in addition to improving the safety of the treatment. However, resistance to EAE induction was comparable between these groups. It could be argued that the Tg4 model, a TCR transgenic mouse, is artificial, thereby raising doubt about the general applicability of the EDI approach. TCR transgenic models provide a unique insight into murine responses, since the precursor frequency of antigen-specific cells is sufficiently high to measure the effect of antigen administration directly. Nevertheless, we have also applied the EDI protocol in non-TCR transgenic models of both cell-mediated and humoral hypersensitivity conditions with success. Furthermore, EDI has been widely used in effective treatment of allergies, including peptide immunotherapy of both cat dander and bee venom allergy^1^. Most importantly, this approach was successfully used in a recent phase 1b study in multiple sclerosis^48^. Arguably, other approaches to immunotherapy, including the use of immune modulating antibodies, would be safer when using an EDI protocol.

Little is known about the mechanisms of antigen-specific desensitization, although it has been practiced for over a century^49^. We have taken a cross-disciplinary approach integrating experimental and computational biology to provide a transcriptome-wide analysis of gene expression associated with effective EDI. The SOM-based approach employed for the analysis of transcriptomics data uses an artificially intelligent algorithm to cluster transcripts within a virtual environment based on changes in the magnitude of expression across all samples. This generates a multidimensional structure, where the more similar gene expression patterns are over time, the closer the transcripts appear in this virtual space. A two-dimensional map, the CPP, is then used to visualize this clustering analysis. This method is superior to conventional data mining techniques, as it does not assume any *a priori* knowledge of the data structure and is less prone to errors than hierarchical clustering methods^50^. The CPP-SOM method not only provides a visual representation of changes in gene expression during the process of therapeutic tolerance induction, but also facilitates inference of dynamic relationships between groups of co-ordinated transcripts, without any assumptions about such interactions. Using this method, we identify marked changes in the cell cycle induced by EDI, repression of transcripts relating to the development of an inflammatory response and induction of transcripts pertaining to regulatory processes. This narration of the biological effects mediated by the sequential transcriptome-wide changes induced during EDI fits with our observations, both *in vitro* and *in vivo*. Expanding on this, EDI was also found to suppress the expression of genes implicated in human autoimmune and inflammatory conditions, including asthma and atherosclerosis.

While analysis of EDI-induced global transcriptional changes provides a significant insight into functionality, we also retain the capacity to focus on the expression of a single gene. This allows us to demonstrate the gradual establishment of a regulatory CD4^+^ T-cell phenotype, characterized by expression of specific negative co-stimulatory molecules and transcription factors, in addition to the regulatory cytokine IL-10. These included transcription factors previously associated with IL-10 expression, including *Maf*, *Ahr* and *Nfil3* (refs 38, 39, 40). The induction of *Il21* expression during EDI is noteworthy, as IL-21 contributes to the IL-27-driven production of IL-10 in murine T cells^38^. *Il21* mRNA was induced by EDI but no cytokine protein was detected in T cells, implying that while *Il10* and *Il21* genes are co-expressed as a result of EDI, there is a further regulatory step that prevents translation of the IL-21 protein. This result also highlights that a range in IL-10-secreting T-cell phenotypes may be induced, influenced by the context of regulatory T-cell differentiation^51^.

Peptide therapy has been shown to induce transient T-cell proliferation followed by anergy^16^,^52^. Our SOM-clustering approach builds on this observation; clusters of genes that were initially induced and then repressed were almost uniquely populated by transcripts encoding proteins involved in the regulation and execution of mitosis. Evidently, these genes were expressed following early treatment with low-dose antigen, but were then repressed at later higher doses, an observation which correlates remarkably with our *in vitro* and *in vivo* observations. The most notable correlation with effective EDI was the induction of a set of negative co-stimulatory molecules including PD-1, LAG-3, TIM-3 and TIGIT. Some of these have previously been associated with T-cell exhaustion^53^, while others have been described as markers of IL-10-secreting Tr1 cells^44^,^54^. By immunolabelling cells from Tg4^Il10/GFP^ reporter mice, we could demonstrate a positive correlation between IL-10 production and the expression of LAG-3, TIGIT, PD-1 and TIM-3. However, expression of these markers was not uniquely restricted to the IL10^+^ population; only 11% of LAG-3^+^ cells were IL-10^+^ and ~50% of Tim-3^+^ or TIGIT^+^ cells were IL-10^+^. These results suggest that while LAG-3 and PD-1 are good markers of the anergic CD4^+^ T-cell population induced by EDI, TIGIT and TIM-3 are better discriminators of IL-10-secreting cells induced by immunotherapy. Recently, CD49b and LAG-3 antibodies were used to identify IL-10-secreting Tr1 cells^44^. CD49b was also found to correlate with IL-10 expression in CD4^+^ T cells from EDI-treated mice; however, within the LAG-3^+^CD49b^+^ population, only 33% of cells were found to express IL-10.

IL-10 plays an essential role in limiting the immune pathology associated with chronic infectious diseases^55^,^56^, and is associated with effective antigen-specific immunotherapy of allergic and autoimmune diseases^1^. The controlled induction of cells secreting IL-10 is clearly a goal for effective immunotherapy of such hypersensitivity conditions. Here we demonstrate the rationale for the use of dose escalation to promote induction of IL-10 and effective immunotherapy. Tolerance induction is achieved safely, and protects animals from autoimmunity in both a prophylactic and therapeutic context. We identify transcription factors and cell surface markers whose expression (both at the mRNA and protein levels) correlates with effective EDI, revealing an insight into the multifaceted process underlying successful immunotherapy. Strategies designed to boost expression of relevant transcription factors, c-Maf and NFIL3, should promote EDI. We also identify a set of negative co-stimulatory molecules whose expression correlates with EDI. This will allow further analysis of the role that IL-10^+^ cells play in immune regulation. With the immunological and transcriptional evidence provided in this study, we anticipate that these molecules can now be investigated as surrogate markers for antigen-specific tolerance induction in clinical trials.

## Methods

### Mice and peptide immunotherapy

TCR transgenic Tg4 and Tg4 Rag-1^−/−^ mice were bred in-house at the University of Bristol, as were B10.PL mice. B6.129S6-Il10^tm1Flv^/J (The Jackson Laboratory)^43^ were crossed with Tg4 mice to generate Tg4^Il10/GFP^ mice. Male and female mice, aged 6–12 weeks, were used. No formal randomization or blinding was undertaken; mice were equally distributed between groups, based on age and sex. All experiments were undertaken with biological and technical repeats, as appropriate for individual assays and defined in figure legends. Data were only excluded in the case of technical or instrumentation failure. Animals were housed under specific pathogen-free conditions, and experiments were performed in accordance with the UK Home Office Project Licence held by D.C.W. and approved by the University of Bristol ethical review committee. For immunotherapeutic tolerance induction, mice were treated s.c. every 3–4 days with 200 μl of MBP Ac1-9[4Y] in phosphate-buffered saline (PBS) at the dose and frequency of dose indicated in individual experiments.

### Peptides

The acetylated native N-terminal murine MBP peptide MBP Ac1-9[4K] (AcASQKRPSQR) and the MBP Ac1-9[4Y] peptide (AcASQYRPSQR) were custom-synthesized by GL Biochem Shanghai.

### Induction and evaluation of EAE

EAE was induced in Tg4 mice by s.c. injection at the tail base of 100 μl of a sonicated emulsion containing equal volumes of CFA and either 1 mg of spinal cord homogenate suspended in PBS or 200 μg of MBP Ac1-9[4K] in PBS. CFA was supplemented with 4 mg ml^−1^ heat-killed *Mycobacterium tuberculosis* (both from Difco). Pertussis toxin (200 ng) (Sigma-Aldrich) was administered i.p. in 500 μl of PBS on days 0 and 2 (refs 17, 57). Alternatively, EAE was induced by adoptive transfer of MBP Ac1-9-specific Th1 cells. Briefly, Tg4 splenocytes were cultured for 5 days with 10 μg ml^−1^ MBP Ac1-9[4K] and 5 ng ml^−1^ rmIL-12 (PeproTech), with 20 U ml^−1^ rhIL-2 (R&D Systems) added after 48 h. Live *in vitro*-polarized Th1 cells (1 × 10^7^) were i.v. transferred. Tg4 Rag-1^−/−^ mice lack nTreg and develop EAE spontaneously^19^. Individual mice were monitored daily for EAE and scored as follows: 0, no disease; 1, flaccid tail; 2, hindlimb weakness and/or impaired righting; 3, hindlimb paralysis; 4, hind and forelimb paralysis; 5, moribund or dead.

### Cytokine protein analysis

MFBI Th1/Th2 Flow Cytomix Multiplex kits (eBioscience) were used to measure the concentration of cytokines in the serum 2 h after s.c. treatment with soluble peptide, while IL-21 and IL-10 Flow Cytomix simplex kits (both eBioscience) were used to assay cell culture supernatant. Fluorescence intensity was measured on a FACS Calibur flow cytometer (BD Biosciences), and data were analysed using FlowCytomix Pro software (eBioscience). Conventional sandwich enzyme-linked immunosorbent assays were performed to quantify cytokine concentration in cell culture supernatant (harvested at 24 h after re-stimulation for IL-2, at 72 h for IFN-γ and IL-10) using matched antibody pairs (all BD Biosciences). IL-2; coating, JES6-1A12 (2 mg ml^−1^), biotinylated, JES6-5H4 (0.5 mg ml^−1^). IFN-γ; coating, R4-6A2 (2 mg ml^−1^), biotinylated, XMG1.2 (0.5 mg ml^−1^). IL-10; coating, JES5-2A5 (2 mg ml^−1^), biotinylated, SXC-1 (0.5 mg ml^−1^). Optical change was measured with a SpectraMax 190 microplate reader (Molecular Devices); cytokine concentration was calculated using Microplate Manager software (Bio-Rad). Intracellular cytokine staining of splenocytes was performed after a 3-h stimulation with phorbol 12-myristate 13-acetate (5 ng ml^−1^) and ionomycin (500 ng ml^−1^) (both Sigma-Aldrich), or a 5-h stimulation with 50 ng ml^−1^ phorbol 12-myristate 13-acetate, 1 μg ml^−1^ ionomycin (for the detection of IL-21) both with GolgiStop (BD Biosciences). Cells were stained with Vβ8-FITC (clone KJ16-133, diluted 1:100) or with fixable viability dye eFluor780 (1:1,000) before surface staining with CD4-Alexa700 (GK1.5, 1:100) and fixation using IC fixation buffer (all from eBioscience). Antibodies for intracellular cytokine staining were diluted in permeabilization buffer; IL-10-allophycocyanin (APC) (JES5-16E3, 1:200), IFN-γ-PerCP-Cy5.5 (XMG1.2, 1:200) (both from eBioscience). For IL-21 staining, 4 μg ml^−1^ recombinant mouse IL-21R Fc chimera (R&D Systems) was used, followed by anti-human immunoglobulin G-PE (Fc γ-specific, 1:250) (eBioscience). Data were collected using an LSR II flow cytometer (BD Biosciences) and analysed using FlowJo software (Treestar). *In vitro*-polarized Th17 cells were used as a positive control for IL-21 production. Briefly, Tg4 splenocytes were cultured for 10 days with 10 μg ml^−1^ MBP Ac1-9[4K], 10 ng ml^−1^ IL-1β, 2 ng ml^−1^ transforming growth factor-β, 25 ng ml^−1^ IL-6 (all cytokines from PeproTech), 10 μg ml^−1^ anti-IL-4 (11b11), 50 μg ml^−1^ anti-IFN-γ (XMG1.2) (both from BioXcell), supplemented with 10 ng ml^−1^ rmIL-23 on day 2 (eBioscience).

### Cell surface intranuclear staining

Splenocytes were stained with fixable viability dye eFluor780 (1:1,000) before staining for surface antigens CD4-A700 (GK1.5, 1:100), CD69-PE-Cy7 (H1.2F3, 1:300), CD62L-PerCP-Cy5.5 (MEL-14, 1:200), LAG-3-PE (C9B7W, 1:200), TIM-3-PE (8B.2C12, 1:200) (all eBioscience) and PD-1-PE-Cy7 (29F.1A12, 1:300), TIGIT-APC (1G9, 1:200), CD49b-PE (HMα2, 1:100) (all Biolegend). For staining of transcription factors and Ki67, cells were then fixed with fixation/permeabilization buffer and antibodies were diluted in permeabilization buffer (both eBioscience); Ki67-PE (SolA15, 1:200), c-Maf-eFluor660 (SYMOF1, 1:200), NFIL3-PE (S2M-E19, 1:200), FoxP3-eFluor450 (FJK-16S, 1:100). Data were collected using an LSR II flow cytometer (BD Biosciences) and analysed using FlowJo software (Treestar).

### Proliferation assays

For *in vitro* intracellular dye dilution proliferation assays, splenic CD4^+^ T cells were isolated from untreated Tg4 mice using a CD4^+^ T-cell isolation kit (Miltenyi Biotec), and labelled with 0.5 μM CFSE (Invitrogen). CFSE-labelled responder CD4^+^ T cells (5 × 10^5^) were then cultured with 1 × 10^6^ irradiated B10.PL splenocytes as APC, 10 μg ml^−1^ MBP Ac1-9[4K] and 5 × 10^5^ CD4^+^ T cells from peptide-treated mice. These had undergone a period of expansion *in vitro* (whole splenocytes cultured with 10 μg ml^−1^ MBP Ac1-9[4K] for 6 days, with 20 U ml^−1^ recombinant human IL-2 added at day 2) before isolation of CD4^+^ T cells using anti-CD4 microbeads (Miltenyi Biotec). After 3 days of co-culture, cells were stained with CD4-APC (GK1.5, 1:100) and 7-AAD (1:400). For *in vivo* intracellular dye dilution proliferation assays, splenic CD4^+^ T cells were isolated from untreated Tg4 CD45.1^+^ mice using a CD4^+^ T-cell isolation kit and were labelled with 2 μM Cell Trace Violet (Life Technologies). Cell Trace Violet-labelled cells (5 × 10^6^) were then injected i.v. into Tg4 CD45.2^+^ recipients. After 24 h, animals were challenged with 80 μg of MBP Ac1-9[4Y]. Spleens were harvested 72 h after challenge. Cells were stained with fixable viability dye eFluor780 (1:1,000), CD4-A700 (GK1.5, 1:100) and CD45.1-PE-Cy7 (A20, 1:300). Cells were analysed using an LSR II (BD Biosciences). Division indices were calculated using FlowJo software (Treestar). For ^3^[H] thymidine proliferation assays, 5 × 10^4^ splenic CD4^+^ T cells were cultured, in triplicate, with 1 × 10^5^ irradiated B10.PL splenocytes as APC and a titration of MBP Ac1-9[4K] concentrations. To measure suppressive capacity, 5 × 10^4^ responder CD4^+^ T cells were cultured with 5 × 10^4^ CD4^+^ T cells from peptide-treated mice, 1 × 10^5^ APC and 0.1 μg ml^−1^ MBP Ac1-9[4K]. Cells were cultured for 3 days and 0.5 μCi of ^3^[H] thymidine was then added to wells for 16 h before measurement with a 1450 Micro-β counter (Wallac).

### Microarray and data analysis

CD4^+^ T cells were isolated using a CD4^+^ T-cell isolation kit (Miltenyi Biotec) from Tg4 Rag-1-deficient mice at six sequential stages of EDI and from PBS-treated controls, 2 h after s.c. challenge with 80 μg of MBP Ac1-9[4Y] (three mice per group). RNA was extracted from CD4^+^ T cells using RNeasy reagents, including DNase treatment (QIAGEN). RNA quality and quantity was assessed using a 2100 Bioanalyzer (Agilent), before biological replicates were pooled. Reverse transcription and amplification was performed at the University of Bristol Genomics Facility using the Ambion WT Expression kit (Applied Biosystems), fragmentation and labelling using GeneChip WT Terminal Labelling and Hybridization kit before hybridization to GeneChip Mouse Gene 1.0 ST arrays (both Affymetrix). Raw expression data were normalized using the Robust Multi-array Averaging algorithm with the quantile normalization method. The quality control showed that an empirical intensity of 200 or less was not reliably discriminated, and thus any values <200 were considered as being technically undetectable. After normalization and pre-processing, probe sets that revealed a two-fold or greater change at four or more treatment points, compared with the PBS-treated control, were identified and considered for further analysis. As such, an expression matrix of 1,893 transcripts at 6 stages of EDI remained for further analysis. A SOM-based method^31^ was used for gene clustering and visualization. Briefly, the gene expression matrix was first trained by SOM with Gaussian kernel. The resulting SOM map was then partitioned into 12 clusters based on topological relationships, without any data structure pre-assumed. The trained SOM map was also visualized by CPPs^32^ to display treatment stage-specific expression changes. For each cluster, enrichment analysis was conducted as previously described^31^ to identify enriched GO-BP (http://www.geneontology.org, performed in December 2012)^33^, MGI-MP (The Jackson Laboratory, http://www.informatics.jax.org/, December 2012)^34^, Reactome (http://www.reactome.org, version 42, December 2012)^35^ and DO (http://disease-ontology.org)^36^ terms. Heatmaps were made using Matrix2png^58^. The expression data are available at NCBI GEO: http://www.ncbi.nlm.nih.gov/geo/query/acc.cgi?acc=GSE46036.

### Real-time PCR

Real-time PCR was performed with QuantiTect SYBR green RT-PCR kits (QIAGEN), using pre-designed QuantiTect Primers (Lag3, QT00113197; Tigit, QT02372314; Pdcd1, QT00111111; Havcr2, QT00120652; Il10, QT00106169; Il21, QT00160678; Maf, QT01063846; Nfil3, QT00265104; Foxp3, QT00138369; B2m, QT01149547 as constitutive control), using an Opticon 2 Real Time Cycler (Bio-Rad). The 2^−ΔΔCT^ method was applied to compare gene expression at the specified stages of EDI versus the PBS-treated control, with or without *in vivo* peptide challenge as indicated.

### Statistical analysis

Statistical analysis was undertaken using tests as indicated, with GraphPad Prism software. Comparison of serum cytokine levels between EDI-treated and 8-μg MBP Ac1-9[4Y]-treated groups was done using a two-way analysis of variance with Bonferroni post-tests. Comparison of Ki67, CD69 and CD62L expression between mice treated with high peptide doses, with or without prior dose escalation, was done using a two-tailed, unpaired *t*-test.

## Additional information

**How to cite this article:** Burton, B. R. *et al*. Sequential transcriptional changes dictate safe and effective antigen-specific immunotherapy. *Nat. Commun.* 5:4741 doi: 10.1038/ncomms5741 (2014).

**Accession codes:** Microarray data was deposited in NCBI GEO, accession code GSE46036.

## Supplementary Material

Supplementary Figures Supplementary Figures 1-5

Supplementary Data 1 Expression matrix of 1,893 regulated transcripts across 6 stages of dose escalation peptide immunotherapy (including 12 gene clusters identified by self-organizing map)

Supplementary Data 2 Enrichment analysis of 12 gene clusters using GO-BP terms
