# Supplementary material for: Sequential transcriptional changes dictate safe and effective antigen-specific immunotherapy
Source: Nat Commun. 2014 Sep 3;5:4741. doi: 10.1038/ncomms5741 (PMC4167604; doi:10.1038/ncomms5741)
Supplement: Supplementary Figures — 1-5 [file ncomms5741-s1.pdf]

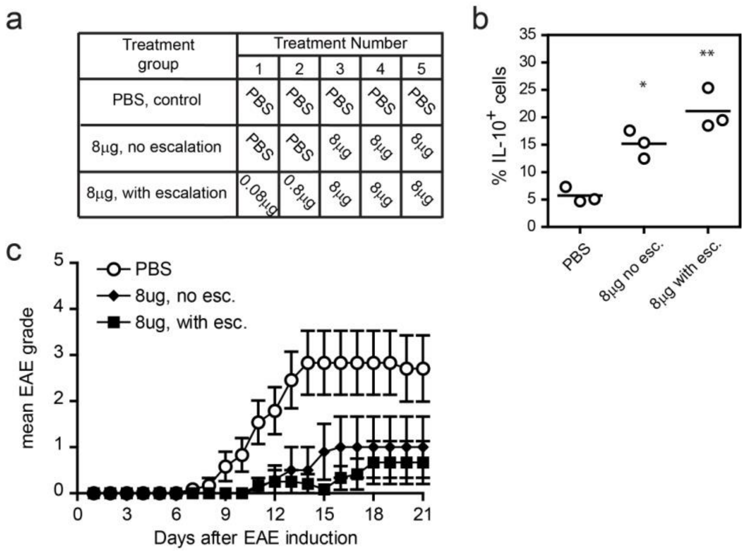

Supplementary Fig. 1 Dose escalation enhances the induction of IL-10^{+} CD4^{+} T cells in addition to minimising the risk of adverse effects during immunotherapy. Tg4 mice were treated s.c. with 3x8μg doses of MBP Ac1-9[4Y], with or without prior dose escalation, as illustrated (a). Splenocytes were cultured for five days with 10μg ml^{-1} MBP Ac1-9[4K] and 20U ml^{-1} IL-2. (b) Scatter plots show the percentage of viable CD4^{+} cells which are IL-10^{+}, horizontal lines show means for each column (*n*=3). One of two experiments. * $P \leq 0.05$, ** $P \leq 0.01$ one-way ANOVA with Bonferroni post-test, comparing peptide-treated groups with PBS-treated control group. (c) Onset and severity of EAE in mice treated as illustrated in (a), immunised with MBP Ac1-9[4K]/CFA and pertussis toxin. Results of two independent experiments are pooled, showing mean disease score ± SEM (PBS group and 8μg, with escalation, both *n*=12. 8μg, no escalation *n*=10).

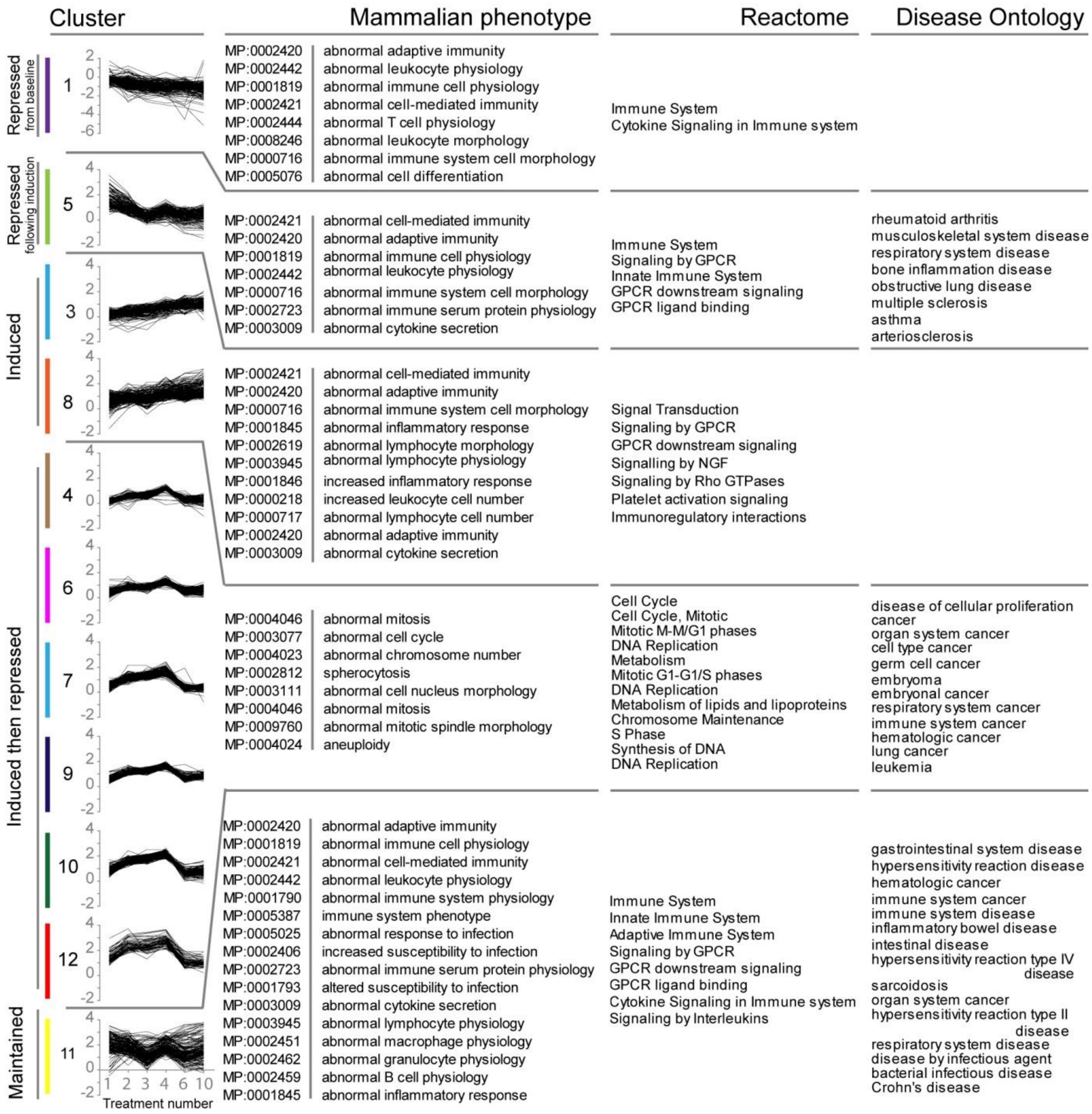

Supplementary Fig. 2 Ontology terms enriched in clusters of co-regulated CD4$^+$ T cell genes during dose escalation immunotherapy. Tg4 Rag-1$^{-/-}$ mice were EDI-treated s.c. with MBP Ac1-9[4Y]. CD4$^+$ T cell transcriptome analysis was undertaken at the indicated treatment stages and transcripts were grouped by two-phase SOM clustering (**see Fig. 6**). Expression pattern and amplitude of expression of transcripts assigned to the twelve color-coded gene clusters described in **Fig. 6b** are here represented by line profiles. Significantly enriched, immunologically relevant annotations from Mammalian Phenotype, Reactome and Disease Ontology databases are shown (FDR $<10^{-5}$ for MP, $<5\times10^{-2}$ for Reactome and DO). Where no terms are shown, no immunologically relevant terms were enriched.

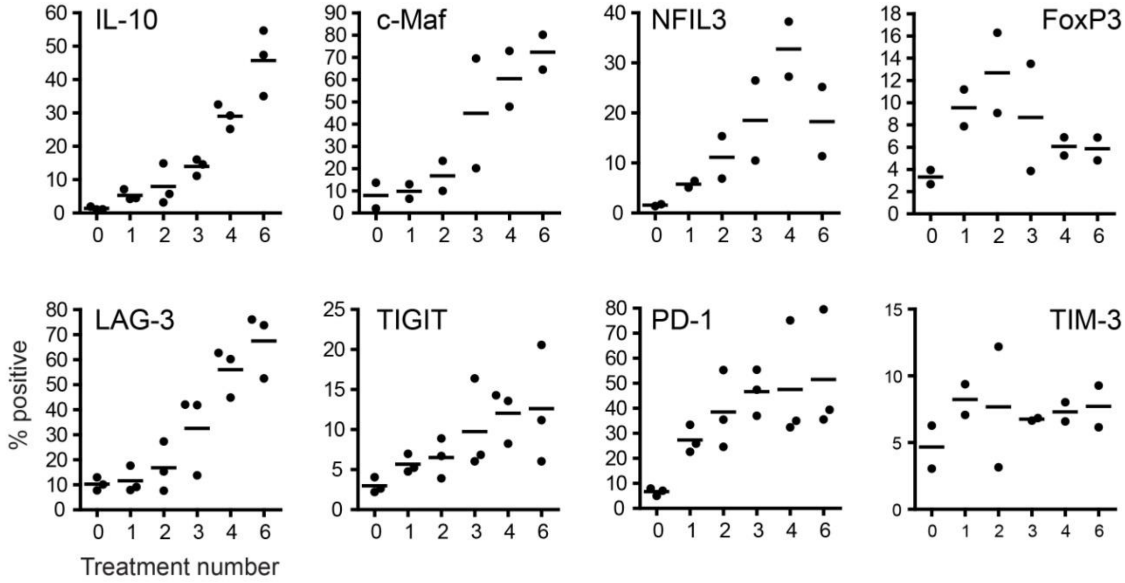

Supplementary Fig. 3 CD4$^+$ T cell signature induced by dose escalation immunotherapy. Tg4 mice were treated s.c. with an escalating dose of MBP Ac1-9[4Y] (0.08μg → 0.8μg → 8μg → 3x80μg). Cells were harvested two hours after peptide challenge *in vivo*. FACS staining of IL-10, c-Maf, NFIL-3, FoxP3, LAG-3, TIGIT, PD-1 and TIM-3 in CD4$^+$ T cells at the indicated stages of treatment. Scatter plots show the percentage of viable CD4$^+$ cells from individual mice which express the signature proteins, horizontal lines show means for each treatment group. Pooled results from two-three independent experiments.

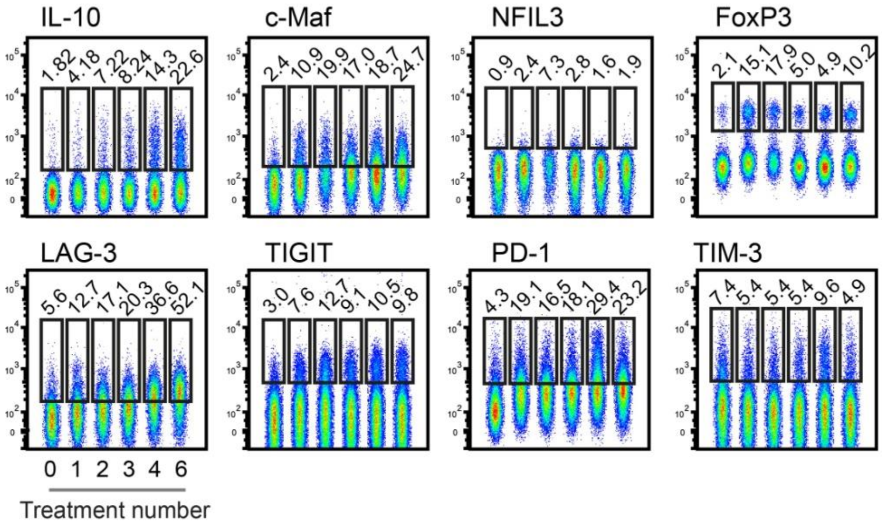

Supplementary Fig. 4 CD4$^+$ T cell signature induced by dose escalation immunotherapy is maintained in resting cells. Tg4 mice were treated s.c. with an escalating dose of MBP Ac1-9[4Y] (0.08µg → 0.8µg → 8µg → 3x80µg). FACS staining of IL-10, c-Maf, NFIL-3, FoxP3, LAG-3, TIGIT, PD-1 and TIM-3 in CD4$^+$ T cells at the indicated stages of treatment. Resting cells were harvested two hours after PBS-challenge *in vivo*, 4 days after the indicated peptide treatment. Data are representative of two-three independent experiments.

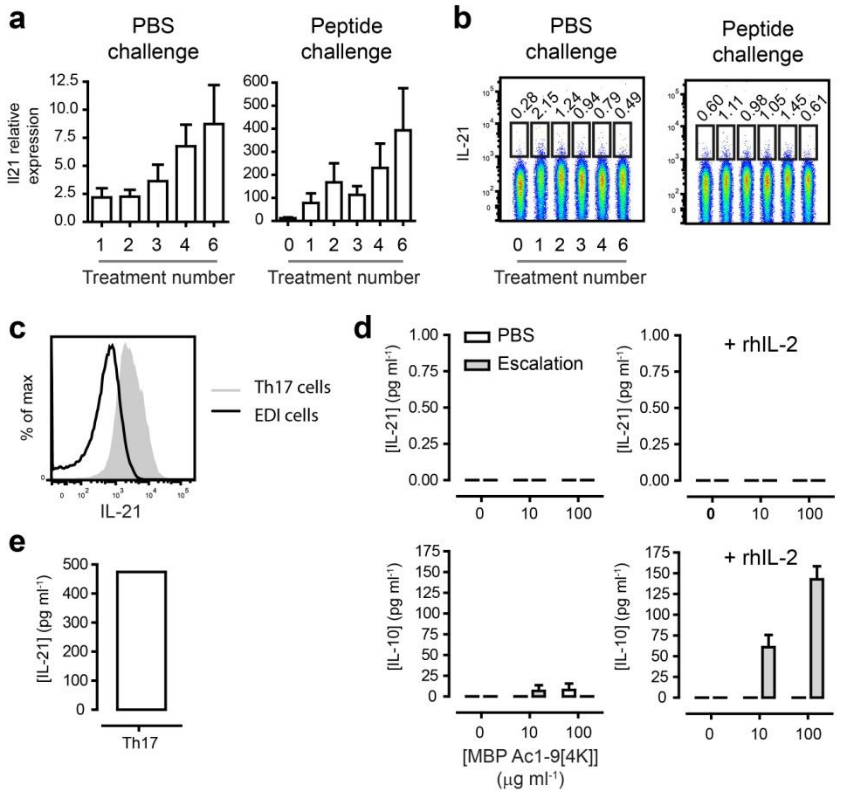

Supplementary Fig. 5 IL-21 mRNA but not protein is produced by $CD4^+$ T cells during dose escalation immunotherapy. Tg4 mice were treated s.c. with an escalating dose of MBP Ac1-9[4Y] (0.08μg → 0.8μg → 8μg → 3x80μg). (a) Quantitative RT-PCR analysis of *Il21* mRNA in $CD4^+$ T cells at the indicated stages of treatment. Graphs show mean expression values + SEM for three replicate experiments, pooled (*n*=3 total). (b) IL-21 expression by $CD4^+$ T cells, detected by FACS at the indicated stages of treatment. (c) FACS-based comparison of IL-21 protein expression by $CD4^+$ T cells from EDI-treated mice (as above) versus Th17-polarised $CD4^+$ T cells as a positive control. Representative of three similar experiments (d) $CD4^+$ T cells from EDI- or PBS-treated mice were re-stimulated in the presence of irradiated APCs and a titration of MBP Ac1-9[4K]. Supernatant was collected after 72 hours to detect IL-21 and IL-10 levels by MFBI. Representative of three similar experiments, error bars show + SEM of at least two independent biological replicates, each assayed in duplicate. (e) IL-21 in the supernatant of Th17-polarised $CD4^+$ T cells, detected by MFBI (positive control). Representative of two experiments, assayed in duplicate.
